# Anti-*Toxoplasma* Effects of *Dracocephalum Polychaetum* Essential Oil

**DOI:** 10.1155/2022/6091834

**Published:** 2022-07-16

**Authors:** Faham Khamesipour, Amirhossein Pourmohammad, Mohsen Jafarian-Dehkordi

**Affiliations:** Faculty of Veterinary Medicine, Shahrekord Branch, Islamic Azad University, Shahrekord, Iran

## Abstract

**Background:**

*Toxoplasma gondii* is a common parasitic disease with a cosmopolitan prevalence, causing severe health problems. Although chemotherapy for toxoplasmosis is readily available, most have side effects.

**Objectives:**

This study assesses the *Dracocephalum polychaetum* essential oil against *T. gondii* activity*. In vitro*, the anti-*Toxoplasma* effects of *D. polychaetum* essential oils with different concentrations were evaluated.

**Methods:**

In the present study, *T. gondii* RH strain tachyzoites were exposed to *D. polychaetum* essential oil, and their viability effect on the parasite was evaluated. The viability test of tachyzoites was performed by using the staining method trypan blue *in vitro*. The inhibitory effect of *D. polychaetum* extract on *T. gondii* RH strain tachyzoites in the Vero cell line was evaluated.

**Results:**

*D. polychaetum* has valuable efficacy *in vitro*, outperforming pyrimethamine and sulfadiazine at 30 and 90 minutes after exposure (*p* < 0.05). *D. polychaetum* essential oil showed anti-*Toxoplasma* activity in the cell line (IC50: 241.7 *μ*g/mL). After *T. gondii*-infected Vero cells had been incubated with different concentrations of the *D. polychaetum* essential oil, their viability decreased in a dose-dependent manner.

**Conclusions:**

In conclusion, *D. polychaetum* extract as an herbal medicine might be a valuable alternative to routine chemotherapy for toxoplasmosis.

## 1. Introduction

Toxoplasmosis is caused by a protozoan parasite called *Toxoplasma gondii* (*T. gondii*) [1, 2]. This protozoan can infect a wide range of warm-blooded vertebrates and humans by living inside the cell. *T. gondii* can cause severe weakness, miscarriage, or even death. The parasite is usually transmitted by drinking water contaminated with cat feces (containing parasitic oocysts) or by eating meat containing uncooked blood and by transfusion of blood from a person infected with the parasite to a healthy person [3, 4].

It is estimated that more than a third of the world's humans have been exposed to toxoplasmosis. About 190,000 people are born with the disease each year [5]. This parasite can spread throughout the host body and uses various tactics to cross the blood-brain barrier and reach the brain [6]. Toxoplasmosis can cause many problems by infecting the fetus by crossing the placenta during pregnancy. Infection in the fetus can manifest itself in the form of growth retardation, mental retardation, eye problems, many other diseases, and fetal death [7]. In the first trimester of pregnancy, if the fetus is infected with the parasite, this infection can cause miscarriage. But if the infection occurs during the second trimester of pregnancy, in that case, this infection can lead to retinochoroiditis and microcephaly in the fetus [8]. If this parasitic infection occurs in the third trimester of pregnancy, it can lead to lymphadenopathy, hepatosplenomegaly, and ocular disorders [8, 9]. Toxoplasmosis is usually asymptomatic in people with sound immune systems [9–11]. However, in people with defective immune systems or underlying diseases such as diabetes, cancer, or people taking corticosteroids, toxoplasmosis causes encephalitis, fever, swollen lymph nodes, fatigue, muscle pain, sore throat, retinitis (inflammation of the retina), and sometimes skin rash [12, 13]. Retinochoroiditis, a symptom of toxoplasmosis, occurs in youth but results from congenital toxoplasmosis [14].

Diagnosis of toxoplasmosis by serological tests is common. However, they are not reliable and can be cited because, as mentioned, there are many anti-*Toxoplasma* antibodies in the blood of one person, and another serological test should be taken a few days later [15, 16].

Toxoplasmosis is usually treated with antiparasitic drugs such as sulfadiazine and pyrimethamine [17], but these drugs have ineffectiveness, resistance, and side effects [18, 19]. Herbal remedies are one of the alternative treatments for many diseases [20–23]. Natural resources, especially medicinal plants, have a high position among the possible new treatments to replace the antiparasitic drugs sulfadiazine and pyrimethamine [24, 25]. Chemical drugs used to treat toxoplasmosis increase serum creatinine and liver enzymes, cause megaloblastic anemia, allergic reactions, and agranulocytosis. In addition, many herbs have traditionally been used to treat toxoplasmosis and have been reported to have. In addition, several papers have demonstrated the pharmacological ability of plant extracts to inhibit the growth of *T. gondii in vitro* [26].

In recent years, the search for new antimicrobial agents has increased, and these agents mainly include plants because about a quarter of the chemical and synthetic drugs currently prescribed are derived from plants or natural sources, which is the case. Studies published in recent years examining natural resources and medicinal plants on protozoan and worm parasites such as *Leishmania, Trypanosoma, Toxoplasma,* and *Echinococcus* have shown that these resources can help treat these diseases [21–25]. Furthermore, the work of Menghini et al. shows that female hemp inflorescences constitute a source of biomolecules with potential pharmacological applications, in particular against parasitosis and infectious diseases [27].

*Dracocephalum polychaetum (D. polychaetum*) belongs to the Lamiaceae family, is used in folk medicine, and contains antioxidant agents [16, 28, 29]. This plant is native to Iran, and it is popularly called Mafro or Badranjboyeh Kermani [29, 30]. This plant species has many uses in Iran due to its many medicinal properties. This plant has been used in the traditional medicine of the Kerman region for a long time due to its pleasant smell and pharmacological properties [29, 30]. No scientific studies have been performed on *D. polychaetum* to replace it with traditional antiparasitic drugs against toxoplasmosis. This study aimed to evaluate the anti-*Toxoplasma* activity of *D. polychaetum* extract *in vitro*. The essential oil of *D. polychaetum* can inhibit *T. gondii* tachyzoites *in vitro* using standard and appropriate methods and was studied to provide scientific data on the anti-*Toxoplasma* properties of this medicinal plant. Therefore, in this study, the therapeutic effect of *D. polychaetum* on *T. gondii* infection was investigated *in vitro*.

## 2. Methods

### 2.1. Ethics Approval

All applicable international and institutional guidelines for conducting the study. The current study was approved by the Ethics Committee of Islamic Azad University, Shahrekord Branch (code: IR.IAU.SHK.REC.1400.066).

### 2.2. Preparation of the Plant and Essential Oil

The aerial part of *D. polychaetum* was prepared, identified, and approved by Kerman province in August 2017, when the plant was fully flowering. In order to prepare the essential oil, the plant was powdered in the dry shade with an electric blender. 100 g of powder was transferred to a 2-liter distillation flask, and 1200 ml of deionized water was added. The essential oil was extracted for 3 hours using a Clevenger essential oil preparation machine. This process was repeated five times to prepare enough essential oil, each time with a new plant. The collected essential oils were then poured on top and dehydrated with anhydrous sodium sulfate, and then stored in a dark closed container away from light and refrigerated [31].

### 2.3. Multiplication, Quantification, and Maintenance of Tachyzoites

Intraperitoneal passages of *T. gondii* RH strain tachyzoites were maintained in Balb/*c* mice and collected in phosphate buffered saline (PBS), pH 7.2, at 3-4-day intervals. The infected mice's peritoneal fluid was collected and centrifuged for 10 minutes at 200 *g* at room temperature. The host cells and detritus were removed by centrifugation. The parasite-containing supernatant was then collected and centrifuged for 10 minutes at 1,000 *g*. The pellet was washed twice, once in PBS at pH 7.2 and again in RPMI-1640 (Gibco, USA) without bovine fetal serum. The trypan blue exclusion technique was used to determine the viability of the parasites 30 to 40 minutes after removal from the peritoneal cavity. Light microscopy and a hemocytometer were used to determine the number of tachyzoites. In a humidified 5 percent CO_2_ incubator at 37°C, the tachyzoites were injected into a 75 ml tissue culture flask containing proliferating Vero cells.

### 2.4. Vero Cells and Evaluation of Cytotoxicity

The kidney cell lines “Vero” were acquired from the National Cell Bank of Iran and were derived from a green monkey kidney (NCBI, Pasteur Institute of Iran, Tehran, Iran). Vero cells were cultured in RPMI-1640 media supplemented with 100 g/ml streptomycin, 100 units/ml penicillin (Gibco, PenStrep15140), 2 ram l‐glutamine, and 10% fetal bovine serum (FBS) at 37°C with 5% CO2 (Bovogen, Australia).

Using 96-well plates, the cytotoxicity of the essential oil was assessed on Vero cells using a modified MTT assay (Sigma Aldrich, USA) with 3(4,5 dimethylthiazolyl) 2,5-diphenyltetrazolium bromide. Vero cells were injected at a concentration of 6 × 10^4^ cells/mL into each well previously holding 100 L growth media. For 24 hours, the cells were incubated at 37°C in an incubator moistened with 5% CO_2_. Following this, the Vero cells were treated with *D. polychaetum* extracts at various concentrations: 1000, 500, 100, 50, 10, and 1 *μ*g/ml. Each concentration was placed in its own 96-well plate. Positive controls included pyrimethamine and sulfadiazine (toxoplasmosis reference medications), whereas the control buffer was RPMI-1640. The supernatant was removed after 24 hours. In each well, 100 *μ*l of MTT PBS solution (5 mg/ml) was mixed with RPMI-1640 in a 1 : 9 ratio. After that, the plate was covered with aluminum foil and placed in a 37°C incubator for 4 hours. After discarding the medium, 100 *μ*l of DMSO was added to each well to dissolve the dark blue MTT formazan salt. A Dynex microplate reader was used to determine the optical density at 570 nm absorbance.

### 2.5. MTT Test and *In Vitro* Infection

For this stage, Vero cells were employed. Exponential growth was used to create the cells. Each well received 3 × 10^5^ parasites/ml, resulting in a total volume of 200 *µ*l. Six hours after inoculation, the infected cells were washed twice with RPMI-1640 FBS free media to eliminate extracellular parasites. After an 18-hour incubation period, 100 *μ*l of RPMI-1640 media with 2% FBS was added to each well, along with various concentrations of essential oil/pyrimethamine and sulfadiazine. The anti- *T. gondii* activity and cytotoxicity of the essential oil were measured in 96-well plates using the thiazolyl blue tetrazolium bromide (MTT) technique (Sigma, St. Louis, MO, USA). The cell line was given the essential oil. A MTT solution was added to the cells after 24 hours, and the presence of purple matrices was determined using a plate reader. All of the data points are the average of three separate trials. The concentration of extracts, controls, and essential oils that effectively inhibited 50% of *T. gondii* tachyzoites was used to calculate the mean inhibitory concentration (IC50). The mean IC50 value for Vero cells compared to the mean IC50 value for *T. gondii* is known as selectivity.

(1) $$\begin{array}{c} \text{SI}\left(\%\right)=\left(\frac{V-\text{IC}50}{T-\text{IC}50}\right). \end{array}$$

*T*‐IC_50_ and *V*‐IC_50_ are the median inhibitory concentrations required to inhibit *T. gondii* and Vero cells, respectively [24].

### 2.6. Tachyzoite Viability by Trypan Blue Exclusion

*In vitro*, a tachyzoite viability test was performed. In 96-well microplates, 45 *μ*l of tachyzoite solution containing 10^6^ cells/ml and *D. polychaetum* essential oil at six different concentrations (1, 10, 50, 100, 500, and 1000 *µ*g) are mixed. The entire mixture was incubated at 37°C. A trypan blue dye exclusion test for tachyzoites is performed under an inverted microscope after 30, 90, and 180 minutes of incubation in 5% CO_2_ at 37°C. The percentage viability of the results was calculated. The positive controls were 96-well plates containing 100 mg/ml of pyrimethamine and sulfadiazine, whereas the negative controls were PBS. After that, the plates were spread out on a glass slide and examined under an optical microscope. Three times the trials were carried out.

### 2.7. Statistical Analysis

ANOVA was used in the statistical analysis using SPSS software (Ver. 18.0). The level of significance was considered to be *p* < 0.05. All data were reported as mean ± SD.

## 3. Results

### 3.1. GC/MS Analysis

**Figure 1:**
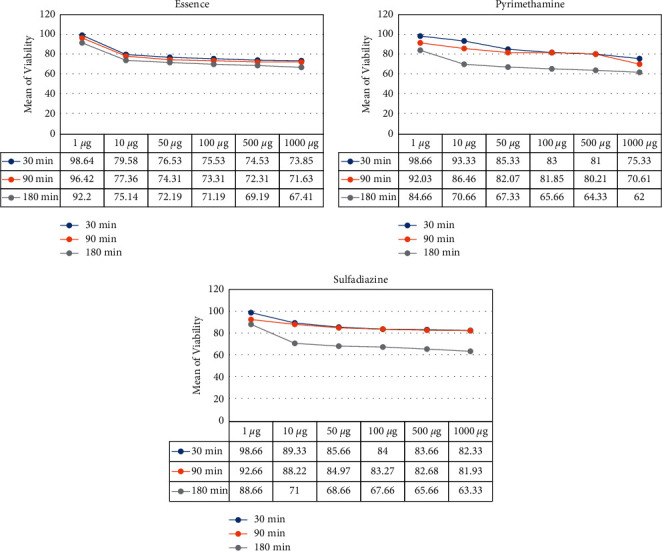
Mean of viability in pyrimethamine, sulfadiazine, and essential oils.

As shown in Table 1, six substances were identified. They represent 88.3% of all the oil. The main substances were methyl cyclogeranete (43.86%), neral (19.98%), and limonene (16.42%).  Figure 1 presents the typical total ion current chromatograms of the essential oil.

### 3.2. MTT Test for Cytotoxicity Activity, *In Vitro* Infection, and Effectors

Table 2 shows the MTT test results for various concentrations of *D. polychaetum* essential oils and negative control cells. *In vitro* tests against *T. gondii* were conducted using a positive control and various concentrations of *D. polychaetum*.

### 3.3. Effects of Essential Oil on Tachyzoite Viability

Six concentrations of *D. polychaetum* essential oil (1, 10, 50, 100, 500, and 1,000 *µ*g) were incubated for 30, 90, and 180 minutes. The vitality of the tachyzoites was determined using trypan blue stain. The parasiticidal impact of essential oil was substantially better than the positive control in all exposure durations, indicating that *D. polychaetum* had adequate effectiveness *in vitro* (Table 3).

**Figure 2:**
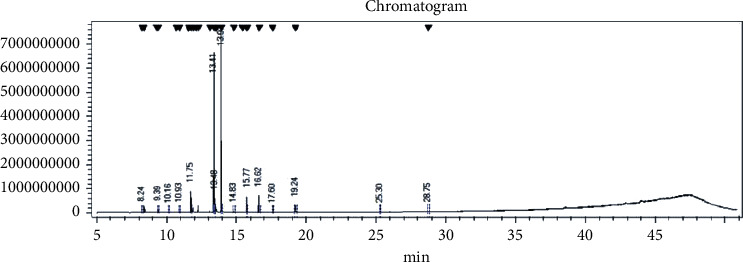
Typical GC-MS total ion current (TIC) chromatograms.

As shown in Figure 2, all three groups were parallel until 90 minutes, and the essential oil group was lower than the two control groups. However, after 90 minutes, this trend was reversed. At 180 minutes, the average viability in the group was that the essential oil was placed above the 2 control groups. *D. polychaetum* essential oil showed anti-*Toxoplasma* activity in the Vero cell (IC50 : 241.7 *μ*g/mL). After *T. gondii*-infected Vero cells had been incubated with different concentrations of the *D. polychaetum* essential oil, their viability decreased in a dose-dependent manner (Figure 2). The viability showed no significant decreases, compared with the control, at all concentrations of the extracts (*p* > 0.05). The mean viability and cytotoxicity between the groups are almost the same.

The viability results between groups with different concentrations and at different times of 30, 90, and 180 minutes showed that in all 3 groups (essential oil and 2 control groups), there was a significant difference between different concentrations (Table 3). There is a measurement in all 3 times. It decreases with increasing concentration to arrive at the highest concentration, i.e., 1000, its lowest value. For pyrimethamine and sulfadiazine, the mean viability at 180 was less than the other two. Simultaneous examination of 3 groups at different times also showed that the interaction between time and group was significant (*p* < 0.001), which indicates that the changes in mean viability depended on the group and the time of measurement.

## 4. Discussion

Numerous studies have been performed to evaluate the anti-*Toxoplasma* activity of plant extracts and essential oils when evaluated *in vitro*. However, valuable and appropriate studies have been performed on natural substances and the identification of essential oils with low toxicity to host cells [24, 25]. It has not been done, and many studies are still needed to determine this issue. The World Health Organization (WHO) recommends using herbal remedies and natural resources for the treatment of toxoplasmosis [32]. People use medicinal plants every day in developing countries because these products are safe, have low side effects, and are available at a low cost [33, 34].

The chemical drugs used to treat this disease have high side effects and common side effects. Numerous studies have been performed to evaluate the anti-*Toxoplasma* activity of plant extracts and essential oils when evaluated *in vitro* [24, 25]. However, valuable and appropriate studies have been performed on natural substances and the identification of essential oils with low toxicity to host cells. It has not been done, and many studies are still needed to determine this issue [24]. Many herbal plants have been introduced as anti-*T. gondii* agents globally. However, there is no research on *D. polychaetum* against toxoplasmosis [18, 19, 35]. The anti-*Toxoplasma* activity of *D. kotschyi* extracts showed that this species had anti-*Toxoplasma* activity [25], so the current work was accomplished [24, 25]. In the current work, we evaluated the efficacy of *D. polychaetum* essential oil on *T. gondii* infection for the first time in Iran. A study conducted by Daryani et al. [35] to investigate the anti-*Toxoplasma* activity of *Sambucus nigra* (Caprifoliaceae) fruit and leaf extract showed that the survival of a group of *T. gondii* parasites exposed to a methanolic extract of *Sambucus nigra* was greater than the other control groups for the first time (30 min), which was not consistent with our study [14, 15, 26].

The selective toxicity of *D. polychaetum* essential oil was near the positive group, but the difference was insignificant. This result was consistent with a previous study [36]. The activity of the essential oil is related to the quality of the active molecules present in it. The presence of active agents in the plant essential oil was determined in the previous studies [37, 38]. Kariminik et al. [16] found that *D. polychaetum* essential oil contains methyl cyclogeranate, limonene, linalool, sabinene, and p-menth-1-en-9-ol. The anti-*Toxoplasma* activity of these active agents was determined too, so the present study was performed. For the first time, we report the activity of *D. polychaetum* essential oil against toxoplasmosis *in vitro*. Supplementary work is needed to identify active compounds of *D. polychaetum* essential oil associated with anti-*Toxoplasma* activity.

## 5. Conclusion

We report for the first time the anti-*Toxoplasma D. polychaetum* activity *in vitro*. This potential is associated with low toxicity to the host cell. Additional work is needed to identify active compounds associated with anti-*Toxoplasma* activity.

## Figures and Tables

**Table 1:** Chemical composition of the oil of *Dracocephalum polychaetum*.

Peaks	Compounds	Molecular formula	Rt (min)	Content (Peak Area %)/%TIC
1	Limonene	C_10_H_16_	9.30	16.42
2	Linalool	C_10_H_18_O	10.70	1.76
3	2-(4-Methylcyclohex-3-en-1-yl) propan-1-ol	C10 H18 O	11.74	4.64
4	Neral	C10H16O	13.40	19.98
5	Sabinene	C10H16	13.56	1.64
6	Methyl cyclogeranete	C10 H16 O	13.93	43.86
7	Minor compounds less than 1%	—	—	11.7
8	Total	—	—	100

Rt, retention time (minutes) of the compounds in column. Peak Area %, percentage of the normalized area which indicates the relative distribution of the compounds in the sample.

**Table 2:** *In vitro* activity and selectivity of essential oil, sulfadiazine and pyrimethamine against *Toxoplasma*.

Tested drugs name	IC_50_		Selectivity index (SI)^a^
	Vero	Vero + *T.gondii*	
Essential oil	386.7	241.7	1.6
Sulfadiazine (positive control)	241.7	111.4	2.17
Pyrimethamine (positive control)	240.9	157.5	1.53

^a^represents the ratio of the IC50 value for Vero cells to the IC50 value for *T. gondii* RH strain.

**Table 3:** The viability between groups with different concentrations and at different times.

Groups	Concentrations (*μ*g)	Time			*P* value
		30 min	90 min	180 min	
Essential oil	1 10 50 100 500 1000	98.64 ± 1.52 79.58 ± 1.00 76.53 ± 1.52 75.53 ± 1.15 74.53 ± 1.15 73.85 ± 1.15	96.42 ± 2.26 77.36 ± 2.63 74.31 ± 2.63 73.31 ± 2.63 72.31 ± 3.11 71.63 ± 2.11	92.20 ± 1.52 75.14 ± 2.08 72.19 ± 2.00 71.19 ± 2.51 69.19 ± 2.51 67.41 ± 2.51	0.631

*P* value		<0.001	<0.001	<0.001	

Sulfadiazine (positive control)	1 10 50 100 500 1000	98.66 ± 0.57 89.33 ± 0.57 85.66 ± 1.52 84.00 ± 2.00 83.66 ± 1.52 82.33 ± 2.08	92.66 ± 0.02 88.22 ± 0.006 84.97 ± 0.03 83.27 ± 0.01 82.68 ± 0.03 81.93 ± 0.00	88.66 ± 1.52 71.00 ± 1.00 68.66 ± 1.15 67.66 ± 1.52 65.66 ± 2.08 63.33 ± 1.52	0.001

*P* value		<0.001	<0.001	<0.001	

Pyrimethamine (positive control)	1 10 50 100 500 1000	98.66 ± 1.52 93.33 ± 2.08 85.33 ± 3.05 83.00 ± 2.00 81.00 ± 2.00 75.33 ± 1.52	92.03 ± 0.01 86.46 ± 0.02 82.07 ± 0.00 81.85 ± 0.01 80.21 ± 0.00 70.61 ± 0.00	84.66 ± 2.51 70.66 ± 2.08 67.33 ± 2.51 65.66 ± 1.52 64.33 ± 2.51 62.00 ± 3.00	0.005

*P* value		<0.001	<0.001	<0.001	

Negative	—	97.00 ± 1.00	95.33 ± 2.51	95.33 ± 1.15	0.958

## Data Availability

The datasets from the present study are available from the corresponding author upon request.

## Abbreviations

*D. polychaetum*: *Dracocephalum polychaetum*
MTT: Thiazolyl blue tetrazolium bromide
*T. gondii*: *Toxoplasma gondii*
IC50: Half-maximal inhibitory concentration
PBS: Phosphate-buffered saline.
